# Traditional Chinese Medicine Syndrome Patterns and Their Association with Hepatitis B Surface Antigen Levels during the Natural History of Chronic Hepatitis B Virus Infection

**DOI:** 10.1155/2018/7482593

**Published:** 2018-10-02

**Authors:** He-Ping Xie, Zhi-Ping Liu, Jiong-Shan Zhang, Min Dai, Ge-Min Xiao, Wei-Kang Wu, Hong-Zhi Yang

**Affiliations:** ^1^Department of Integrative Chinese and Western Medicine, The Third Affiliated Hospital of Sun Yat-sen University, Guangzhou 510630, Guangdong Province, China; ^2^Guangdong Key Laboratory of Liver Disease Research, The Third Affiliated Hospital of Sun Yat-sen University, Guangzhou 510630, Guangdong Province, China; ^3^Institute of Integrated Traditional Chinese and Western Medicine, Sun Yat-Sen University, Guangzhou 510080, Guangdong Province, China; ^4^Department of Biochemistry and Molecular Biology, School of Basic Medicine, Gannan Medical University, Ganzhou 341000, Jiangxi Province, China

## Abstract

The aim of this study is to investigate traditional Chinese medicine syndrome (TCMS) patterns and their association with hepatitis B surface antigen (HBsAg) levels during the natural history of chronic hepatitis B virus infection (CHB). Patients were categorized according to the phase of CHB, as follows: immune tolerance (ITP); immune clearance (ICP); low or nonreplication (LRP); reactivation (RAP); hepatic cirrhosis (HC); and primary liver cancer (PLC). TCMS patterns were classified among the following types: spleen-kidney deficiency (SKD); liver-qi depression (LQD); damp-heat in liver-gallbladder (LGDH); liver-kidney deficiency (LKD); and blood stasis blocking collateral (BSBC). HBsAg levels and other serological indicators were quantified for all patients and their association with TCMS was statistically analyzed and determined. Two hundred and eighty-nine patients with CHB were included. During the natural history of CHB, TCMS patterns were statistically different among the different phases (*P < *0.001). The most frequently occurring syndromes among the six progressive phases were SKD, LGDH, LKD, LGDH, BSBC, and LGDH, respectively. The predominant patterns in the inactive stage (ITP + LRP), active stage (ICP + RAP), and late or advanced stage (HC + PLC) were SKD (31%), LGDH (51.8%) and BSBC (34.4%), respectively. Median HBsAg levels were also statistically different among the five patterns of TCMS (*P <* 0.001). The highest HBsAg levels were observed in SKD (4.48 log_10_ IU/mL). Medium levels were in LQD (3.91 log_10_ IU/mL) and LGDH (3.90 log_10_ IU/mL). The lowest HBsAg levels were in LKD (3.60 log_10_ IU/mL) and the second lowest levels in BSBC (3.81 log_10_ IU/mL). In addition, HBsAg levels in LKD and BSBC were significantly lower than those in SKD, LQD, and LGDH (*P* < 0.05 or 0.001). TCMS was altered during the natural history of CHB and correlated with HBsAg titers. This study could provide further insight into the therapy of CHB.

## 1. Introduction

Due to high risk of developing acute or chronic hepatic failure and hepatocellular carcinoma (HCC), chronic hepatitis B virus (HBV) infection (CHB) remains a heavy burden and substantial challenge to global public health [1–3]. With the widespread use of anti-HBV agents in the past decades, particularly interferon-based regimens and nucleos(t)ide analogs, much progress has been made on the therapy of CHB. However, current therapy is still limited to the suppression of viral DNA replication, and prolonged use of nucleos(t)ide analogs induces more viral mutation [4]. In addition, there are some other predicaments of the current therapies, including expensive costs, side effects, and relapse after discontinuation of antiviral therapy. Traditional Chinese medicine (TCM), which features syndrome differentiating and a holistic concept, may provide an effective alternative therapy to solve such problems in anti-HBV treatment.

With advancement in understanding of CHB pathogenesis, the natural history of CHB has been recently classified into the following four phases: immune tolerance phase (ITP); immune clearance phase (ICP); low or nonreplication phase (LRP); and reactivation phase (RAP)[5–7]. Their respective clinical diagnoses are asymptomatic carrier, hepatitis B e antigen- (HBeAg-) positive hepatitis, inactive carrier, and HBeAg-negative hepatitis. These typical four phases do not include hepatic cirrhosis (HC) or primary liver cancer (PLC). It is well known that patients with CHB, especially in high endemic areas such as our country, have high risk of developing cirrhosis and HCC, with cumulative lifetime incidences of 41.5% and 21.7%, respectively [8]. Therefore, the progressive course of HBV infection to CHB, HC, and PLC represents a trilogy of CHB-associated liver diseases.

Syndrome differentiation is the well-recognized essence of TCM, and as such identification of syndrome patterns is key to preventing and treating a disease by this approach [9, 10]. The traditional Chinese medicine syndrome (TCMS) pattern distribution of the active state of CHB has been developed. Although, the TCMS pattern of chronic HBV carriers was elucidated in ITP [9, 11–13], until now no comprehensive study has systematically investigated these consecutive phases and the TCMS patterns during the complete natural history of CHB remain unclear.

As the first identified and main marker of HBV infection, hepatitis B surface antigen (HBsAg) is positively correlated with DNA and partly reflects the content of covalently closed circular DNA (cccDNA) [14–16]. Our previous studies found that there was no significant correlation between DNA load and the TCMS types in ITP patients [17]. But, it is still unknown whether there is a correlation between HBsAg and the types of TCMS or not. HBsAg is considered an important predictor of clinical and therapeutic outcomes of HBV infection, and the most direct and powerful clinical evidence of HBV clearance is the absence of HBsAg [15, 18–21]. However, the correlation between HBsAg and TCMS patterns is also yet to be studied.

In this study, we investigated TCMS patterns and their association with HBsAg levels during the complete natural history of CHB, including the four typical phases, HC, and PLC.

## 2. Materials and Methods

### 2.1. Ethics Statement and Informed Consent Statement

The study was reviewed and approved by the Ethics Committee of the Third Affiliated Hospital of Sun Yat-sen University (Approval No. 2013:2-60). All patients voluntarily joined this study, and patients who accepted liver biopsy provided written informed consent.

### 2.2. Sample Collection

This study was conducted in patients with CHB, from the Department of Integrative Chinese and Western Medicine at The Third Affiliated Hospital of Sun Yat-sen University (Guangzhou, China). All patients were classified among the six groups, based on four typical phases (ITP, ICP, LRP, or RAP) and two late or advanced stages (HBV-related HC or PLC) in the course of CHB. The diagnostic criteria for the four typical phases were based on the 2012 European Association for the Study of the Liver [22]. Diagnosis of HC and PLC was comprehensively judged by clinical manifestations, liver function, alpha-fetoprotein level, liver imaging findings, and the Chinese Guideline of Prevention and Treatment for Viral Hepatitis published in 2000 [23]. The detailed criteria of the phase classification of consecutive phases in the natural history of CHB are outlined in Table 1.

Exclusion criteria for the study included (1) coinfection with hepatitis C virus, hepatitis D virus or human immunodeficiency virus, (2) coexistence of alcoholic, metabolic or autoimmune liver disease, (3) concomitant serious disease in heart, lung or kidney, (4) history of mental illness, (5) no signs of cirrhosis or PLC but having a history of taking anti-HBV medication, (6) diagnosis of nonHBV-associated liver cirrhosis, or (7) diagnosis of secondary liver cancer.

### 2.3. Parameters

The following parameters were recorded for each patient: TCMS; demographical information; status and levels of serum liver biochemical markers and serum (qualitative) HBV markers and serum (quantitative) HBsAg, HBeAg, and HBV DNA levels.

### 2.4. Serum HBsAg Titers and HBV DNA Load

Serum HBV markers were quantitatively measured by enzyme-linked immunosorbent assay (ELISA). Serum HBsAg titers were measured with a commercial detection kit (Abbott Architect assay; Abbott Diagnostics, Wiesbaden, Germany) following the manufacturer's instructions. The dynamic range of the kit was 0.05-250 IU/mL. If titers were more than 250 IU/mL, the samples were diluted 1:500 or 1:1000 using the HBsAg Manual Diluent (Abbott Diagnostics). HBV DNA was qualitatively detected by fluorescence quantitative PCR assay (Da-An Gene, Sun Yat-Sen University, China).

### 2.5. Standards and Performance of TCMS Differentiation

TCMS of patients were differentiated among five types, with reference to the viral hepatitis diagnostic standards defined in December 1991 by the Internal Medicine Hepatopathy Committee of the Chinese Traditional Medicine Association [24] and our previous study [25]; these five types included spleen-kidney deficiency (SKD), liver-qi depression (LQD), inner damp-heat retention (LGDH), liver-kidney deficiency (LKD), and blood stasis blocking collateral (BSBC). All TCMS pattern differentiation was checked firstly by an attending doctor and then verified by a chief physician. Some patients had two or more TCMS patterns, both in this study and our previously published study [9]. In this study, we did not design treating interventions and only considered the relationship between TCM syndromes and HBsAg. Therefore, in the course of the study, we mainly considered and recorded the patient's primary syndrome.

Standards of TCMS were based on the Guiding Principles for Clinical Research on New Drugs of Traditional Chinese Medicine (published in 2003)[26], the sixth edition of Diagnostics of Traditional Chinese Medicine [27], and the clinical experience of the TCM doctors who specialized in preventive and therapeutic strategies of infectious diseases. The differentiating standards for the classification of the five syndrome types are listed in Table 2.

### 2.6. Statistical Analysis

All serum statistical data are presented as medians with 10%–90% confidence intervals (CIs). Between-group comparisons were carried out by analysis of variance test (ANOVA) with either Mann-Whitney U (in nonparametric test for univariate comparisons) or Kruskal–Wallis (for multivariate comparisons). Categorical data were tested using the *χ*^2^ test. The correlation between two variables was analyzed by Pearson's correlation coefficient (*r*). All the statistical analyses were carried out using the SPSS statistical software, version 19.0 (IBM Corp., Armonk, NY, United States).

## 3. Results

### 3.1. Patients' General Characteristics Based on the Natural History of CHB

Two hundred and eighty-nine patients with CHB were enrolled in the study and divided into the six groups, representing ITP (*n* = 70), ICP (*n* = 50), RAP (*n* = 33), LRP (*n* = 72), HC (*n* = 19), and PLC (*n* = 45). Twenty-six patients with alanine aminotransferase (ALT) < 2 × upper limit of normal (ULN) were classified as ITP, based on their histological activity indices (HAIs) of liver biopsy not indicating significant inflammation (G ≤ 1). Seventeen patients with ALT > 1.5–2 × ULN were classified into ICP, according to their HAIs of liver biopsy indicating significant inflammation (G ≥ 2). Nine patients with ALT < 2 × ULN, but whose ALT elevations were not associated with HBV infection, were classified as LRP.

Two hundred and twenty-five patients in the four typical phases did not receive antiviral treatment. For the late or advanced stage groups, 11 cases of HC and 23 cases of PLC were treated with oral antiviral agents. The overall data on patients' demographics, serum liver biochemical markers, serum qualitative HBeAg status, and HBsAg and HBV DNA quantitation are presented in Table 3. All data for the six groups reached the threshold for statistically significant difference (*P *< 0.001), indicating that the method of dividing natural history was accurate and reliable.

### 3.2. TCMS Patterns during the Natural History of CHB

**Figure 1:**
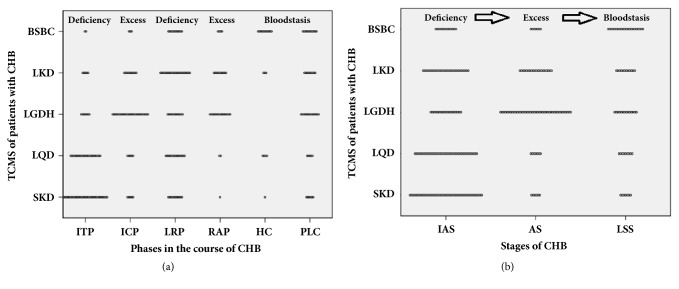
**2-D dot plots of TCMS patterns in six phases (a) and three stages (b) during natural history of CHB.** Each 2-D dot represents an individual pattern. AS: active stages set; BSBC: blood stasis blocking collateral; CHB: chronic hepatitis B virus infection; HC: hepatic cirrhosis; IAS: inactive stages set; ICP: immune clearance phase; ITP: immune tolerance phase; LGDH: damp-heat in liver-gallbladder; LKD: liver-kidney deficiency; LQD: liver-qi depression; LRP: low or nonreplication phase; LSS: late or advanced stages set; PLC: primary liver cancer; RAP: reactive phase; SKD: spleen-kidney deficiency; TCMS: traditional Chinese medicine syndrome.

During the natural history of CHB infection, the TCMS patterns varied significantly between patients in different phases of CHB (*P* < 0.001; Figure 1(a)). The two predominant patterns in ITP were SKD (47.1%) and LQD (32.9%). The patterns were similar between ICP and RAP, as the most frequent two patterns in both phases were LGDH (54% and 48.5%, respectively) and LKD (20% and 30.3%, respectively). In LRP, the most frequent pattern was LKD (31.9%), and the other patterns were nearly equal. In HC, the most frequent pattern was BSBC (57.9%), and in PLC the most frequent two patterns were LGDH (31.1%) and BSBC (24.4%).

### 3.3. Distribution of TCMS Patterns in Three Sets

In the four typical phases of CHB, ITP and LRP are the inactive states, while ICP and RAP are the active states. Additionally, HBV-associated PLC often presents after HC. Hence, we combined the six groups into three sets, namely, the inactive stage set (IAS), the active stage set (AS), and the late or advanced stage set (LSS). As shown in Figure 1(b), the patterns of TCMS were also significantly different between patients in the three sets of CHB (*P* < 0.001). The first predominant pattern in IAS was SKD (31%), in AS was LGDH (51.8%), and in LSS was BSBC (34.4%).

### 3.4. Distribution of Serum HBsAg Titers in Five Patterns of TCMS

**Figure 2:**
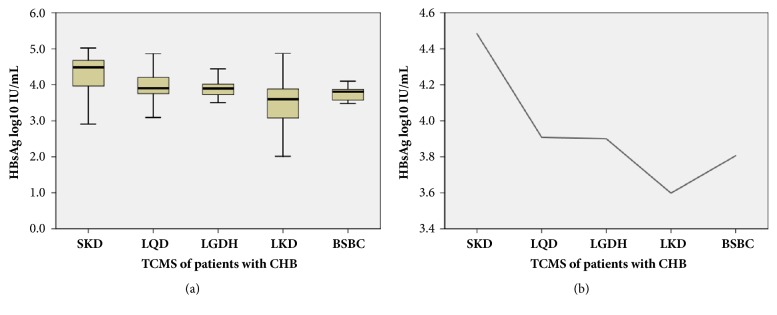
**Distribution of HBsAg titers in the five TCMS patterns of patients with CHB.** (a) Box plot of the distribution of HBsAg titers in the five TCMS patterns. Bars represent 95% confidence interval of the median. (b) Line plot of medians of HBsAg titers in the five TCMS patterns. CHB: chronic hepatitis B virus infection; HBsAg: hepatitis B surface antigen; TCMS: traditional Chinese medicine syndrome.

The distributive profile of serum HBsAg titers from this study is shown in Figure 2(a). HBsAg titers differed significantly among the five patterns of TCMS (*P* = 0.001; univariate ANOVA comparison). The medians of HBsAg titers are plotted in Figure 2(b), showing that the highest median titer among the five patterns was in SKD (4.48 log_10_ IU/mL), moderate titers were in LQD (3.91 log_10_ IU/mL) and LGDH (3.90 log_10_ IU/mL), relative lower titer was in BSBC (3.81 log_10_ IU/mL), and the lowest was in LDK (3.60 log_10_ IU/mL).

Further statistical analysis of the HBsAg titers in Figure 2(a) showed that those of SKD were higher than those in LQD (*P *< 0.05) and more so than those in the other three patterns (LGDH, LKD and BSBC) (*P *< 0.001). The HBsAg titers in LQD and LGDH were higher than those in LKD and BSBC (*P *< 0.05). No differences were found between LQD and LGDH, nor between LKD and BSBC (*P *> 0.05).

### 3.5. Correlation between Serum HBsAg and HBV DNA and HBeAg in TCMS Patterns of CHB

**Figure 3:**
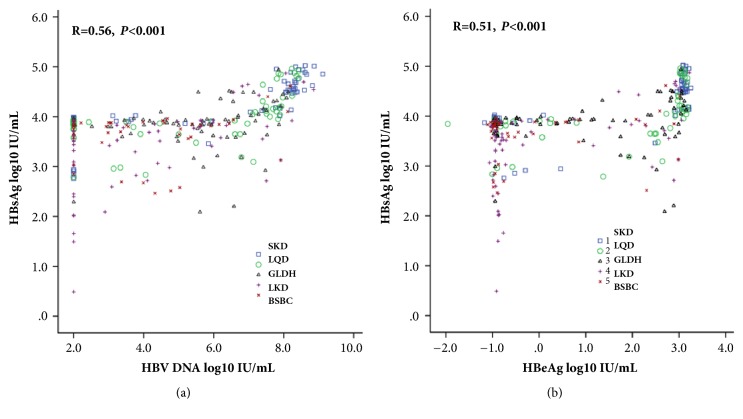
**Correlations between serum HBsAg titers and HBV DNA (a) and HBeAg (b) in TCMS patterns of CHB.** CHB: chronic hepatitis B virus infection; HBeAg: hepatitis B e antigen; HBsAg: hepatitis B surface antigen; HBV DNA: hepatitis B virus DNA; TCMS: traditional Chinese medicine syndrome.

As shown in Figure 3, HBsAg was correlated with HBV DNA and HBeAg levels (*P *< 0.001). Furthermore, in each pattern of TCMS, HBsAg and HBV DNA levels still showed a positive correlation in patients in the SKD (*r* = 0.80,* P* < 0.001), LQD (*r* = 0.58,* P *< 0.001), and LKD (*r* = 0.53,* P *< 0.001) groups, but no positive correlation in patients in the LGDH or BSBC group. In each pattern of TCMS, HBsAg, and HBeAg levels showed a positive correlation in patients in the SKD (*r* = 0.71,* P *< 0.001) and LQD (*r* = 0.52,* P *< 0.001) groups, but no positive correlation in patients in the LGDH, LKD, or BSBC group.

## 4. Discussion

As CHB is a highly dynamic pathological process, its trilogy has been used to represent the progressive course across hepatitis, cirrhosis, and PLC since the last century. In recent decades, hepatitis B has been further divided into HBeAg-positive hepatitis and HBeAg-negative hepatitis [5]. And, in the last 5 years, the natural history of CHB has been classified into four typical phases (ITP, ICP, LRP and RAP). At the same time, TCM initially attached importance to the ever-moving endpoint of these phases. According to this concept, TCMS may change throughout the course of CHB, as was partly demonstrated in our previous study [9]. Therefore, in this study, we tried to further explore the transformation of TCMS during the serial phases of the natural history of CHB in patients.

As shown in Figure 1, the distribution of the TCMS pattern was significantly different throughout the natural history of CHB. For the six phases, the pattern followed as SKD, LGDH, LKD, LGDH, BSBC, and LGDH. Our previous study also found that SKD was the most frequent pattern in ITP patients [9].

Both excess syndrome caused by deficiency and deficiency syndrome caused by excess can be used to explain the change of the nature or mechanism of a disease in TCM. As far as TCMS of CHB is concerned, those results presented in Figure 1(a) serve as the main clue towards elucidating the change of this disease's nature. From ITP to ICP, the key point of the transformation of TCMS was deficiency causing excess, i.e., the deficiency of spleen and kidney turned into damp-heat in liver and gallbladder. From ICP to LRP, the key point was excess causing deficiency,* i.e.* damp-heat in liver and gallbladder turned into the deficiency of liver and kidney. From LRP to RAP, the key point again was excess syndrome caused by deficiency, i.e., the deficiency of liver and kidney turned into damp-heat in liver and gallbladder.

After reconstruction of the six phases into three bundled sets, the predominant pathogenic factors in the inactive, active and late or advanced stage sets of patients were deficiency (spleen, liver,and kidney), excess (damp-heat) and blood stasis, respectively. Deficiency was the main nature of CHB in ITP and LRP, that is, in the inactive stage sets. A likely reason for this finding is that the patients had a congenital Yang deficiency in spleen and kidney, which also may be the most likely explanation for their being infected by HBV. Another possible explanation is that the HBV infection led patients to the Yang deficiency in spleen and kidney. The factor causing Yin deficiency of liver and kidney in LRP was possibly due to damp-heat consuming Yin. Conversely, excess was the main nature of the disease in ICP and RAP, that is, the active stage sets. Their common reason could be the intensive struggle between the vital and evil qi. Obviously, the reason for explaining blood stasis as the leading pathogenic factor in HC and PLC is that HC and PLC often developed in late-stage CHB.

In summary, the pattern, nature and pathogenic factor varied with the difference of phases or stages. Phases and stages of the natural history of CHB could be considered as indicators of differentiating TCMS. Besides that, the results of this study also verified that TCMS was the dynamic manifestation of the progressive process of disease. This study had significant implications for understanding the natural history of CHB from the TCM perspective, and might provide the joints for the combination of Chinese and Western medicines. Generally, the inactive-stage TCM therapeutic principle should mainly strengthen vital qi, including reinforcement of spleen and kidney Yang in ITP and nourishment of liver and kidney Yin in LRP. And, in active-stage, TCM should mainly eliminate evil qi, clearing damp-heat in liver and gallbladder. Finally, in HC and PLC, TCM should mainly activate blood circulation to dissipate blood stasis.

Studies have also revealed that serological HBsAg titers have a positive association with HBV DNA level, as well as with intrahepatic cccDNA level [20, 28–30]. Furthermore, the quantitative test of HBsAg is less expensive than of HBV DNA. Therefore, dynamic surveillance of HBsAg levels has been used as a key factor in calculating and assessing response to antiviral treatment [31, 32]. Exciting findings were obtained from a very recent study which showed that higher HBsAg levels were associated with development of cirrhosis and HCC comparatively and that lower serum HBsAg levels were associated with a higher rate of spontaneous HBsAg seroloss [21, 33]. What is more, higher HBsAg levels might mean more possible reactivation in LRP patients.

In the future, it may be a meaningful but challenging effort to develop a therapy that will allow for further decrease in HBsAg levels, even down to complete absence, especially in those patients whose HBV DNA has been successfully restrained. TCM, with the Yin-Yang theory, pays attention to reinforcing the vital essence and strengthening the primordial qi. It is no doubt that TCM is very suitable for providing subsequent therapy to those patients who have successfully achieved suppression of HBV replication but who still have high HBsAg titers.

HBsAg titers change with development of the natural history in CHB [34], but its concrete mechanisms are still undiscovered. In addition, no study of TCM to date has sought to explore the related understanding of HBsAg; hence, one of goals of this study was to develop the TCM knowledge of HBsAg.

As shown in Figure 2, HBsAg levels varied among the five types of TCMS in CHB patients. HBsAg levels in SKD, LQD, and LGDH were higher than in LKD and BSBC. Moreover, levels were highest in SKD and lowest in LKD, but quite similar in LQD and LGDH. So, the evidence obtained in the present study supported that high HBsAg titers are associated with SKD and low titers with LKD. Figure 3 further demonstrates the association among HBsAg, HBeAg, HBV DNA, and TCM syndromes. All of the above suggests that HBsAg levels might serve as a microindicator for differentiating TCMS in patients with CHB.

HBsAg is not only a pathogenic factor and its titers also are affected by functions of human organs. Thus, the general mechanisms underlying the connection between HBsAg and TCMS may be two-sided. As far as patients are concerned, an abnormal state of physiological functions or substances not only provides opportunities for HBV infection, but also affects the process and outcome of the HBV infection, obviously including the production of HBsAg. Conversely, HBV will disturb and harm the physiological functions and substances of patients since HBV can integrate into the host genome [35, 36].

The different HBsAg titers also represent different phases or stages of CHB. However, the detailed theories of the interactions among HBV, its markers, and TCMS are not clear. TCMS has its own objective bases and essence [37, 38]. The present study implicated HBsAg as likely being one of the bases of TCMS for patients with CHB. Hence, further investigation into the nonHBV-associated substances or markers of TCMS, by using the methods of molecular biology, will also be necessary and may help reveal the mechanisms of the interactions among HBV, HBsAg, and TCMS.

As a whole, we found positive correlation among the levels of HBsAg, HBV DNA, and HBeAg. Moreover, this correlation existed in SKD and LQD, but not in LGDH and BSBC. Integrating different HBsAg levels in each pattern of TCMS, the present general results suggest that differences in the pattern of TCMS might result from the variation of virological features and viral life cycle during the natural history of CHB. We could not, however, exclude the influence of inherent constitutions in patients. In future research a matched-pair study should be considered, wherein each selected case will be compared with a healthy volunteer without HBV infection.

Our main objective has been achieved in this study. Our findings support the hypothesis that TCMSs vary and associate with HBsAg throughout CHB. There is, however, a particular shortcoming of this study that must be considered when interpreting these findings—the insufficiency of sample size in some phases, specifically the RAP and LC phases, which precluded our ability to analyze the connection of HBsAg and TCMS in each different phase of CHB separately. In order to obtain more powerful evidence to more definitively identify HBsAg, HBeAg, and HBV DNA as indicators of TCMS differentiation, prospective studies are now necessary, as they will allow for assessments of the associations between HBsAg and TCMS in every phase or stage of CHB.

In conclusion, this study found significant differences of TCMS patterns throughout the natural history of CHB. The findings from this study have significant implications for understanding the natural history of CHB from the TCM perspective and might provide the theoretical joints for combining Chinese and Western medicines. Furthermore, HBsAg titers were found to vary among different TCMS patterns of CHB patients. Discovering the association of HBsAg titers and TCMS patterns contributes to our overall understanding the HBV viral life cycle and interaction between pathogenic factors (HBV and HBsAg) and vital essence in the TCM perspective. Knowing baseline HBsAg levels in different TCMS patterns may provide further insight into the therapy of CHB, integrating Chinese and Western medicines.

## Figures and Tables

**Table 1:** Criteria of the classification of phases in the natural history of CHB.

**Phase**	**ALT** **∗** ** in U/L**	**HBeAg status**	**HBV DNA in IU/mL**	**Liver imaging**
ITP	< ULN	+	> 10^7^	normal
ICP	≥ 2 × ULN	+	> 2000	nonHC and nonPLC
LRP	< ULN	-	< 2000	normal
RAP*∗∗*	≥ 2 × ULN	-	> 2000	nonHC and nonPLC
HC	any	+/-	any	cirrhosis
PLC	any	+/-	any	PLC

^*∗*^Patients with ALT < 2 × ULN but no significant inflammation (G ≤ 1) in liver biopsy would be put into ITP. Patients with ALT > 1.5–2 × ULN but significant inflammation (G ≥ 2) in liver biopsy would be put into ICP. Patients with ALT < 2 × ULN, but whose ALT elevations were not HBV-associated, would be put into LRA. The normal ALT was 40 U/L. *∗∗*Every patient in RAP had at least one recorded event of hepatitis activity, in order to avoid patients that belong in ICP with HBV precore mutation in G1896A from being mistakenly categorized into RAP. ALT: alanine aminotransferase; HBV DNA: hepatitis B virus DNA; HBeAg: hepatitis B e antigen; HC: hepatic cirrhosis; ICP: immune clearance phase; ITP: immune tolerance phase; LRP: low or nonreplication phase; PLC: primary liver cancer; RAP: reactive phase; ULN: upper limit of normal. “-”: negative; “+”: positive.

**Table 2:** Differentiating standards of the five patterns of TCMS.

**Syndrome**	**Main points of differentiation**
LQD	Irritability, depression, fullness or pain in the hypochondrium, slight yellow coat on the tongue, taut pulse
LKD	Dry eyes and throat, feverish sensation in the chest and palms, lumbar debility, scanty coating on the tongue, fine and rapid pulse
LGDH	Fatigue, bitter mouth, dark urine (jaundice), unsmooth bowel movement, yellow and oily coat on the tongue
SKD	Poor appetite, urinary frequency or enuresis, loose stool, cold feeling and weakness in the lumbar spine and knee, white slippery coating on the tongue, moderate pulse
BSBC	Needle-pricking sensation and pain, dark face, dark tongue, fine and sluggish pulse

BSBC: blood stasis blocking collateral; LGDH: damp-heat in liver-gallbladder; LKD: liver-kidney deficiency; LQD: liver-qi depression; SKD: spleen-kidney deficiency; TCMS: traditional Chinese medicine syndrome.

**Table 3:** Patient characteristics based on the six phases in the natural history of CHB.

**Phase**	**ITP,** ***n* = 70**	**ICP,** ***n* = 50**	**LRP,** ***n* = 72**	**RAP,** ***n* = 33**	**HC,** ***n* = 19**	**PLC,** ***n* = 45**	***P***
**Sex, M/F**	36/34	42/8	54/18	29/4	17/2	38/7	< 0.001
**Age in ** **yr**	27 (21-61)	34 (31-37)	38 (35-40)	41 (37-45)	48 (41-56)	54 (45-57)	< 0.001
**ALT, ** **U/L**	38 (33-40)	183 (80-236)	24 (22-28)	175 (88-261)	53 (42-64)	55 (43-67)	< 0.001
**AST, ** **U/L**	27 (25-28)	121 (89-152)	25 (24-27)	97 (61-133)	82 (52-111)	91 (65-116)	< 0.001
**HBV DNA, log**_**10 **_ **IU/mL**	7.99 (7.89-8.21)	6.77 (6.57-7.22)	2.00 (2.00-2.00)	5.61 (5.09-5.68)	4.42 (2.66-5.48)	3.42 (3.00-4.01)	< 0.001
**HBsAg, ** **log**_**10 **_ **IU/mL**	4.51 (4.41-4.60)	3.93 (3.71-4.15)	3.71 (3.57-3.81)	3.86 (3.83-3.88)	3.81 (3.68-3.85)	3.83 (3.80-3.86)	< 0.001

Age is listed as median and min.-max. ALT, AST, HBV DNA, and HBsAg are expressed as the median and 10%-90% confidence interval. ALT: alanine aminotransferase; CHB: chronic hepatitis B virus infection; HBV DNA: hepatitis B virus DNA; HBsAg: hepatitis B surface antigen; HC: hepatic cirrhosis; ICP: immune clearance phase; ITP: immune tolerance phase; LRP: low or nonreplication phase; PLC: primary liver cancer; RAP: reactive phase.

## Data Availability

The data used to support the findings of this study are available from the corresponding author upon request.

## Supplementary Materials

Supplementary Materials In order to investigate traditional Chinese medicine syndrome (TCMS) patterns and their association with hepatitis B surface antigen (HBsAg) levels during the natural history of chronic hepatitis B virus infection (CHB), patients were categorized according to the disease phase or stage of CHB, as follows: immune tolerance (ITP); immune clearance (ICP); low or nonreplication (LRP); reactivation (RAP); hepatic cirrhosis (HC); and primary liver cancer (PLC). The patients' TCMS patterns were classified among the following types: spleen-kidney deficiency (SKD); liver-qi depression (LQD); damp-heat in liver-gallbladder (LGDH); liver-kidney deficiency (LKD); and blood stasis blocking collateral (BSBC). HBsAg levels and other serological indicators were quantified for all patients. All clinic data were recorded in the supplementary materials.
